# Oncologic outcome and recurrence patterns of clinical stage IB and IIA cervical cancer: A large retrospective analysis of a tertiary reference center

**DOI:** 10.1002/ijgo.70366

**Published:** 2025-07-15

**Authors:** Osman Aşıcıoğlu, Sinan Ateş, Füsun Varol, Tülin Deniz Yalta, Cenk Sayın

**Affiliations:** ^1^ Department of Obstetrics & Gynecology, Faculty of Medicine Trakya University Edirne Turkey; ^2^ Department of Pathology, Faculty of Medicine Trakya University Edirne Turkey

**Keywords:** lymph node metastasis, parametrial involvement, radical hysterectomy, recurrence

## Abstract

**Objective:**

To evaluate the outcomes and recurrence patterns and define the parameters that can help to predict high‐risk patients among our 20‐year clinical early‐stage IB–IIA cervical cancer patients treated with traditional (non‐nerve sparing) radical surgery.

**Methods:**

This retrospective cohort study included 158 patients who had undergone traditional (non‐nerve sparing) radical hysterectomy with pelvic lymph node dissection ± paraaortic lymph node dissection with clinical early‐stage IB–IIA cervical cancer between January 2005 and July 2024 in our referral center. Descriptive statistics, survival analyses, and recurrence sites were evaluated. Furthermore, we researched independent risk factors for parametrial involvement and lymph node metastasis in these patients.

**Results:**

The median follow‐up time was 112 (1–288) months. The 5‐year disease‐free survival and overall survival rates in this study were 84% and 89%, respectively. There were two (1.2%) patients with local recurrence, four (2.4%) with regional, and seven (4.4%) patients with distant recurrence. According to multivariate logistic regression analyses, the only independent risk factor for regional and distant recurrences was lymph node metastasis.

**Conclusion:**

Traditional (non‐nerve sparing) radical surgery appears to be safe and necessary to improve local control and decrease the local recurrence rate in clinical early‐stage IB–IIA cervical cancer patients. We observed that lymph node metastasis was the only independent risk factor for regional and distant recurrences. However, there was no independent risk factor for local recurrence in patients with clinical stage IB–IIA cervical cancer.

## 1 INTRODUCTION

Cervical cancer is the second most common cause of death from gynecological cancers, despite the increasing primary and secondary prevention methods available .^1^ Curative surgical treatment, which includes radical type III hysterectomy and pelvic‐paraaortic lymph node dissection, is preferred in clinical early‐stage FIGO IB and IIA cervical cancers (ESCC). The effectiveness of curative surgical treatment is crucial for oncological outcomes (overall survival [OS], disease‐free survival [DFS]) and recurrence patterns.^2^, ^3^, ^4^, ^5^, ^6^ Traditionally, a radical type III hysterectomy procedure (non‐nerve sparing radical hysterectomy) includes the removal of all uterine, parametrial, and paracervical tissue. The radical hysterectomy technique has advanced in recent years, and a new nerve‐sparing technique has been described and performed to reduce complications such as bladder atony, sexual dysfunction, and urinary fistula in the postoperative period.^7^ In fact, in recent years, studies have reported that a simple hysterectomy is sufficient in low‐risk (tumor size <2 cm and limited stromal invasion) cervical cancers.^8^ As the application of radical surgery decreases and nerve‐sparing surgery increases, the biggest concern has been the increase in local recurrence. The micro invasion in parametrial tissues, perineural spread, and the inadequate (minimum 5 mm negative tumor cell surgical margin) removal of parametrial and vaginal tissues are to be among the most important causes of local recurrence.^9^, ^10^ Because the reported microscopic or macroscopic parametrial involvement (PI) in ESCC is 5.4%–25%, sufficient parametrectomy is vital in these women.^11^, ^12^ Furthermore, nerve‐sparing surgery may increase local recurrence rates through perineural invasion and perineural spread, which can cause local recurrence.^10^ Recently, the SCCAN study reported that in the existence of a tumor diameter of 2–4 cm, traditional (non‐nerve sparing) radical hysterectomy has a superior 5‐year disease‐free survival rate compared to nerve‐sparing radical hysterectomy, but there is no difference in the patterns of local or distant recurrences.^13^ However, many other studies and meta‐analyses have stated that nerve‐sparing radical hysterectomy has no disadvantage in regard to OS and DFS rates.^14^ In summary, the complications of radical surgeries on one side and the safety of less radical and nerve‐sparing surgeries especially in patients with microscopical PI on the other side constitute a dilemma in cervical cancer curative surgical treatment strategies, and further investigation is necessary.

In our study, we aimed to investigate the parameters that can help to predict high‐risk patients and to evaluate the outcomes and recurrence patterns in our 20‐year ESCC patients treated with traditional (non‐nerve sparing) radical surgery.

## 2 MATERIALS AND METHODS

This retrospective cohort study consists of 158 patients with ESCC of FIGO 2018 cervical cancer (histopathologically confirmed as human papillomavirus‐related cervical cancer) who underwent traditional (non‐nerve sparing) radical hysterectomy with pelvic lymph node dissection ± paraaortic lymph node dissection between January 2005 and July 2024 at Trakya University, Faculty of Medicine, Department of Obstetrics and Gynecology. The study was approved by the Trakya University Non‐Interventional Scientific Research Ethics Committee (TUTF‐GOBAEK No: 2025/26). It was actualized in accordance with the ethical standards of the Helsinki Declaration. İnformed consent was obtained from all women. All operations were performed by expert gynecologists. Clinical staging was performed by speculum inspection and bimanual pelvic and rectovaginal examination. Transvaginal ultrasonography (TvUsg) was used to evaluate the tumor diameter. When the parametrial invasion was suspected, a pelvic magnetic resonance imaging (MRI) was performed, and if involvement was not reported, surgery was performed. If there was an obvious parametrial invasion on examination or obvious lymph node metastasis (LNM) on MRI evaluation (‐MRI reported LNM was described as greater than 20 mm in the maximal shortaxis diameter measured on T2 weighted imaging in transverse axial planes), the patient was referred for chemoradiotherapy. In patients who were operated on until 2018, retrospective restaging was performed based on clinical examination and radiological evaluation (TvUsg and MRI) findings on patients' files, using the new FIGO 2018 staging system. The exclusion criteria of our study were: patients receiving radiotherapy or neoadjuvant chemotherapy as primary therapy, patients with other primary gynecological malignancies besides cervical cancer, patients with coincidentally diagnosed cervical cancer subsequent to simple hysterectomy for another indication, patients with histopathologic diagnosis of nonsquamous or nonadenocarcinoma, and patients with insufficient records.

All patient data were obtained retrospectively from patients' files (age, body mass index, and menopausal status). All surgical and biopsy specimens were interpreted by expert gynecological pathologists. Tumor dimensions, the histopathological type of the primary tumor, the lymphovascular space invasion (LVSI) status, the depth of stromal invasion (DSI), the existence of PI, and lymph node (LN) status were recorded. DSI was defined as the measurement of the tumor from the epithelial–stromal junction of the adjacent most superficial epithelial papilla to the deepest point of invasion, and stromal invasion deeper than two‐thirds of the cervical wall was defined as deep stromal invasion. LVSI was described as the existence of tumor cells inside the capillary lumens of either the lymphatic or microvascular drainage systems within the primary tumor. The presence of tumor cells seen from the surgical margin of pathological material is described as a positive surgical border. Vaginal involvement was defined as the identification of tumor cells elsewhere in the vaginal region. OS was identified as the time from the date of primary surgery to death or the latest observation. DFS was identified as the time from the date of primary surgery to the detection of recurrence or the latest observation. The recurrence patterns were classified according to recurrence sites. Recurrences in the vaginal stump and the paravaginal region were defined as local recurrence, regional recurrence was defined as recurrence in the pelvic lymph node region, and distant recurrence was defined as recurrences in another area of the body outside the pelvis, including the para‐aortic lymph nodes, distant organs, bone, muscle, and peritoneal cavity.

Adjuvant treatment decision was determined by the gynecological oncology council as adjuvant radiotherapy to patients with intermediate risk factors (LVSİ [+], large tumor size [>4 cm], DSI) and chemoradiotherapy to patients with high‐risk factors (lymph node metastasis (+), presence of parametrial invasion confirmed by pathology report, and positive surgical margins).^15^, ^16^ All patients underwent a standard follow‐up every 3 months in the first 2 years, every 6 months in 3–5 years, and annually after 5 years. In the follow‐up period, chest X‐ray and either MRI, computed tomography, or positron emission tomography scans were performed once a year, and upon any suspicion of recurrence based on bimanual pelvic examination, symptoms, and an increase in serum tumor markers more than two times (cancer antigen 12–5, carcinoembryonic antigen, cancer antigen 19–9, and squamous cell carcinoma antigen).

The *χ* ^2^‐test was used for categorical variables, and Student's *t*‐test was used for normally distributed continuous variables. Survival analysis based on the Kaplan–Meier method and results were compared using the log‐rank test. We used Fisher's exact test to identify an association between the recurrence site and clinicopathological parameters. Cox regression analysis was used to determine factors affecting survival, and results were presented as hazard ratios. We performed logistic regression models for univariate and multivariate analyses based on the correlation between PI, LNM, and preoperative and postoperative clinicopathological features. Survival curves were plotted using the Kaplan–Meier method. The results were considered to be statistically significant at *p* < 0.05. All statistical analyses were applied using IBM SPSS Statistics for Windows software (version 24.0; IBM Corp).

## 3 RESULTS

**FIGURE 1:**
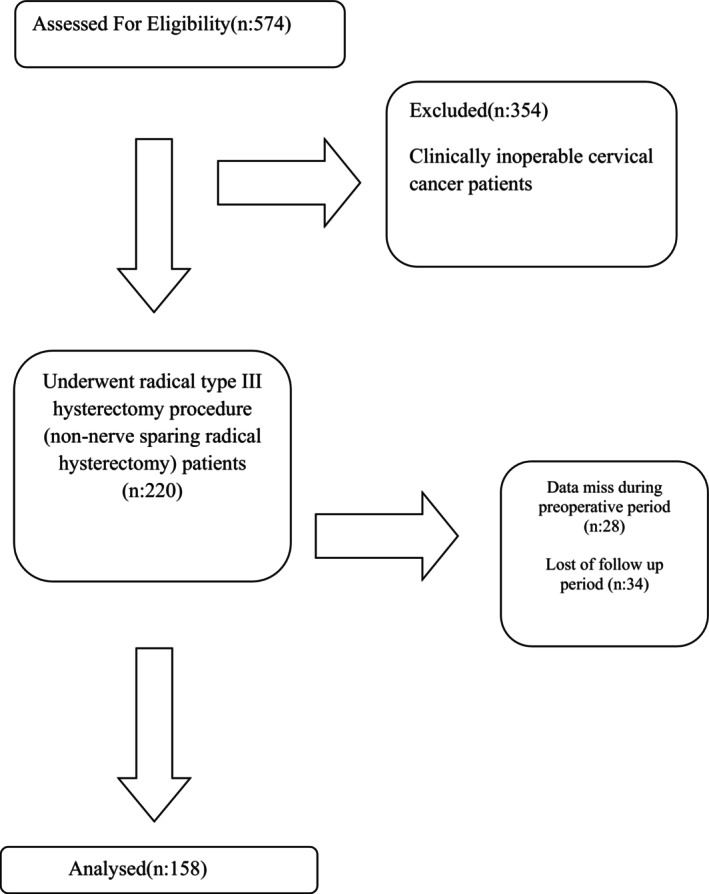
Flow diagram of research.

A total of 158 ESCC patients were identified for the research (Figure 1). The general characteristics and oncological outcomes of the 158 patients are shown in Table 1. The median age of the patients at surgery was 57.5 years. Approximately 58.2% of the patients were in clinical stage IB1. The rate of PI was 8.9%, and the rate of LNM was 8.3%. The surgical margin positivity was detected in only one patient. The median follow‐up time was 112 (1–288) months. The 5‐year DFS and OS rates in this study were 84% and 89%, respectively. There were two (1.2%) patients with local recurrence, four (2.4%) with regional recurrence, and seven (4.4%) patients with distant recurrence (Table 1). Nine cases died of cervical cancer in the study period. Site‐specific recurrence pattern risk factors are presented in Tables 2 and 3. In the univariate analysis, clinical FIGO stage, vaginal involvement, and lymph node metastasis were found to be related to local recurrence. Regional recurrence was also interrelated with clinical FIGO stage, parametrial involvement, and lymph node metastasis. Distant recurrence also tended to be associated with clinical FIGO stage, vaginal involvement, PI, and LNM (Table 2). According to multivariate logistic regression analysis, only LNM was found to be an independent risk factor for regional and distant recurrences (Table 3).

**TABLE 1:** Clinical features of patients with clinical stage IB–IIA cervical cancer.

Age [years, mean ± SD]	57.5 ± 10.7
Postmenopozal status, [*n* (%)]	101 (63.9)
BMI [kg/m^2^, mean ± SD]	28.1 ± 5.7
FIGO stage, [*n* (%)]	
IB1	92 (58.2)
IB2	42 (26.5)
IB3	13 (8.2)
IIA1	8 (5.0)
IIA2	3 (1.8)
Histology, [*n* (%)]	
Squamous	117 (74.0)
Adenocarcinoma	26 (16.4)
Mixed	15 (9.5)
Tumor diameter [mm, mean ± SD]	3.2 ± 0.9
LVSI (+), [*n*, (%)]	79 (50)
Parametrial involvement, [*n*, (%)]	14 (8.9)
Vaginal invasion, [*n*, (%)]	39 (24.7)
Positive surgical margin, [*n*, (%)]	1 (0.6)
Lymphadenectomy, [*n*, (%)]	
Pelvic	24 (15.2)
Pelvic + Para‐aortic	134 (84.8)
Lymph node metastasis, [*n*, (%)]	13 (8.3)
Depth stromal invasion, [*n*, (%)]	64 (40.5)
Adjuvant treatment, [*n*, (%)]	
Radiotherapy	52 (32.9)
Chemotherapy	9 (5.7)
Chemoradiotherapy	10 (6.3)
Recurrence, [*n*, (%)]	
Local	2 (1.2)
Regional	4 (2.4)
Distant	7 (4.4)
OS [months, median (min‐max)]	55.2 (1–288)
DFS [months, median (min‐max)]	53.1 (1–288)
5‐year OS (%)	89
5‐year DFS (%)	84
5‐year local control rate (%)	99
Follow‐up [months, median (min‐max)]	112 (1–288)

Abbreviations: BMI, body mass index; DFS, disease‐free survival; LVSI, lymphovascular space invasion; OS, overall survival.

**TABLE 2:** Clinical and pathological characteristics of patients having site‐specific recurrence.

Factors	Patients, [*n*, (%)]	Local recurrence, [*n*, (%)]	*p*	Regional recurrence, [*n*, (%)]	*p*	Distant recurrence, [*n*, (%)]	*p*
Age (years), [*n*, %)]							
≤ 50	59 (37.3)	1 (1.7)	0.710	1 (1.7)	0.600	2 (3.4)	0.620
>50	99 (62.7)	1 (1)		3 (3.0)		5 (5.1)	
Clinical FIGO stage, [*n*, (%)]							
IB1	92 (58.2)	0	0.007	0	0.001	0	<0.001
IB2	42 (26.5)	0		1 (2.4)		2 (4.8)	
IB3	13 (8.2)	1 (7.6)		1 (7.7)		3 (23.1)	
IIA1	8 (5.0)	1 (12.5)		1 (12.5)		1 (12.5)	
IIA2	3 (1.8)	0		1 (33.3)		1 (33.3)	
Histology, [*n*, (%)]							
SCC	117 (74.1)	2 (1.7)	0.399	3 (2.6)	0.965	4 (3.4)	0.297
AC‐ASC	41 (25.9)	0		1 (2.4)		3 (7.3)	
Vaginal invasion, [*n*, (%)]							
Positive	39 (24.7)	2 (5.1)	0.013	2 (5.1)	0.234	4 (10.3)	0.042
Negative	119 (75.3)	0		2 (1.7)		3 (2.5)	
Parametrial involvement, [*n*, (%)]							
Positive	14 (8.9)	0	0.657	2 (14.3)	0.003	3 (21.4)	0.001
Negative	144 (91.1)	2 (1.4)		2 (1.4)		4 (2.8)	
LVSI, [*n*, (%)]							
Positive	79 (50)	1 (1.3)	0.990	3 (3.8)	0.311	2 (2.5)	0.246
Negative	79 (50)	1 (1.3)		1 (1.3)		5 (6.3)	
Lymph node metastasis, [*n*, (%)]							
Positive	13 (8.3)	1 (7.7)	0.030	2 (15.4)	0.002	4 (30.8)	<0.001
Negative	145 (91.7)	1 (0.7)		2 (1.4)		3 (2.1)	
Depth stromal invasion, [*n*, (%)]							
Positive	64 (40.5)	1 (1.6)	0.783	3 (4.7)	0.155	5 (7.8)	0.088
Negative	94 (59.5)	1 (1.1)		1 (1.1)		2 (2.1)	

Abbreviations: AC, adenocarcinoma; ASC, adeno‐squamous cell carcinoma; LVSI, lymphovascular space invasion; SCC, squamous cell carcinoma.

**TABLE 3:** Multivariate analysis of risk factors for site‐specific recurrence patterns.

	Local	*p*	Regional	*p*	Distant	*p*
	OR (95% CI)		OR (95% CI)		OR (95% CI)	
Vaginal invasion	2.60 (1.10–6.10)	0.094	1.80 (0.80–3.90)	0.455	1.90 (1.50–2.30)	0.501
Parametrial involvement	1.70 (0.90–3.30)	0.125	2.00 (1.20–3.40)	0.302	2.20 (1.50–3.10)	0.091
Lymph node metastasis	3.50 (1.40–8.70)	0.088	5.60 (1.10–27.10)	0.020	9.10 (2.20–40.80)	0.011

Abbreviations: CI, confidence interval; OR, odds ratio.

**FIGURE 2:**
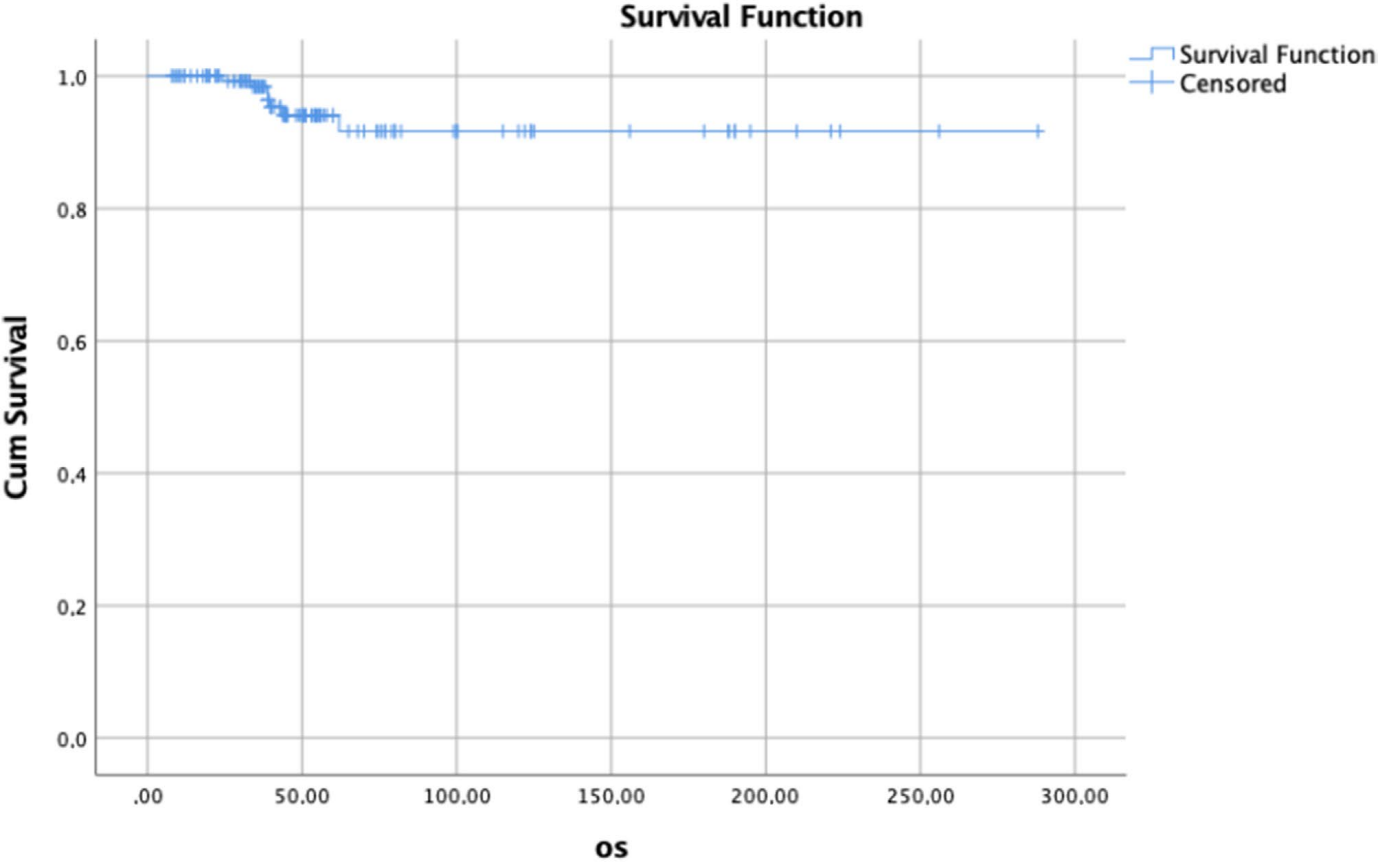
Kaplan–Meier estimate of overall survival of the overall population.

**FIGURE 3:**
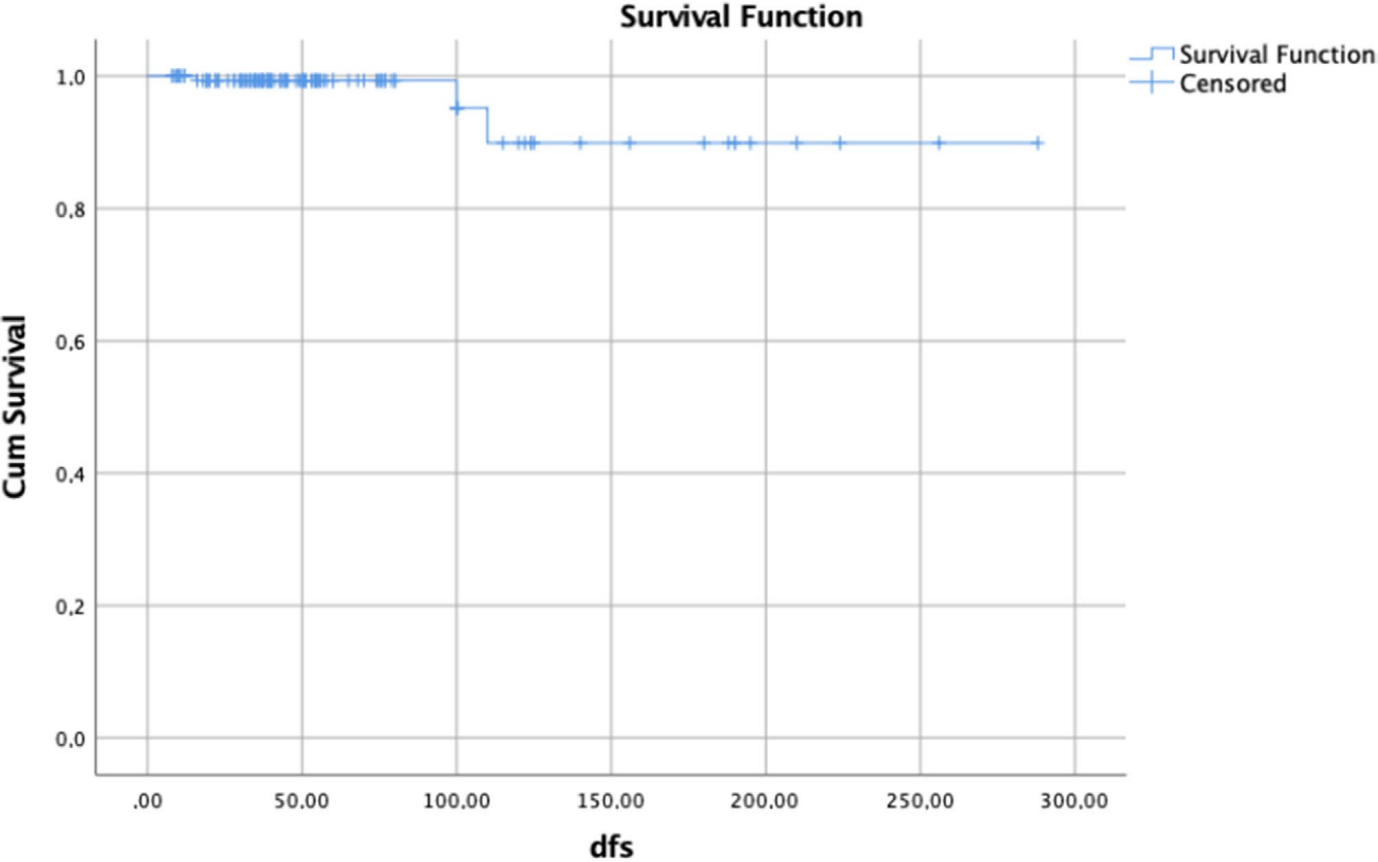
Kaplan–Meier estimate of disease‐free survival of the overall population.

**FIGURE 4:**
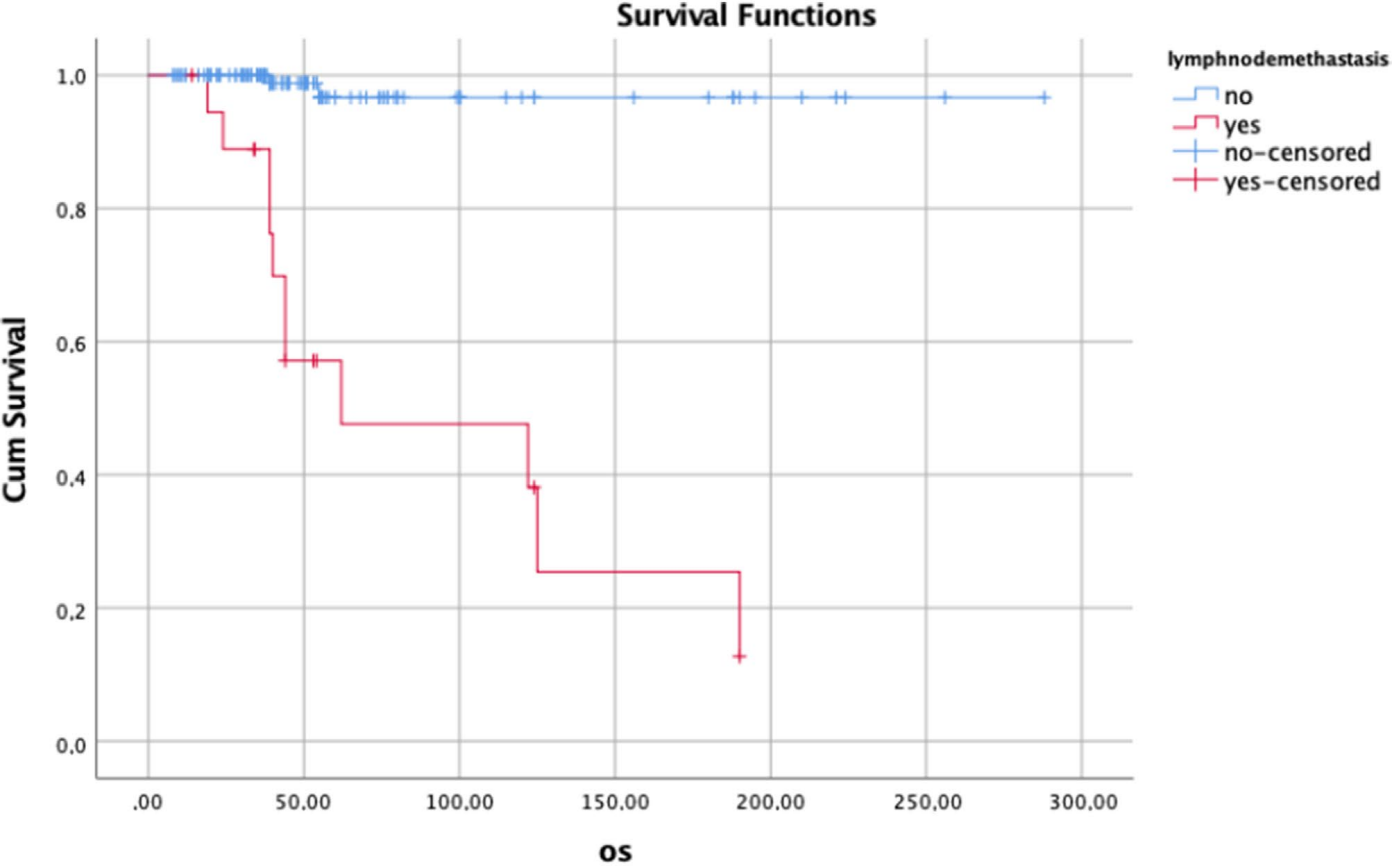
Survival outcomes according to lymph node metastases.

The results of univariate and multivariate analysis for OS are shown in Table 4. In the univariate analysis, vaginal involvement, PI, LNM, and development of recurrence (local, regional, or distant) were related to short OS. Multivariate logistic regression analysis revealed that LNM, local, regional, and distant recurrences were independent predictors of OS (Table 4). Figures 2, 3, 4 show survival curves.

**TABLE 4:** Univariate and multivariate analysis for overall survival in patients with clinical stage IB–IIA cervical cancer.

	Univariate	*p*	Multivariate	*p*
	Hazard ratio (95% CI)		Hazard ratio (95% CI)	
Vaginal invasion	5.10 (2.00–11.50)	0.023	3.00 (1.90–4.40)	0.086
Parametrial involvement	6.00 (2.00–17.80)	0.030	3.60 (2.00–5.80)	0.077
LVSI	4.40 (2.20–8.70)	0.105	3.90 (3.30–4.40)	0.188
Lymph node metastasis	8.50 (3.50–21.40)	0.008	7.10 (3.60–13.90)	0.040
Depth stromal invasion	5.20 (2.90–9.90)	0.090	4.60 (3.60–5.80)	0.139
Local recurrence	8.80 (2.80–21.20)	0.004	7.00 (3.50–14.10)	0.003
Regional recurrence	9.90 (5.00–19.20)	0.002	9.60 (4.10–23.20)	0.001
Distant recurrence	12.50 (2.50–61.10)	<0.001	11.90 (3.00–34.20)	<0.001

Abbreviations: CI, confidence interval; LVSI, lymphovascular space invasion; OR, odds ratio.

The results of univariate and multivariate analysis for PI are shown in Table 5. In the univariate analysis, vaginal involvement, tumor diameter, LNM, and DSI were found to be related to preoperative PI. In the multivariate analysis, the only independent risk factor for PI in the preoperative period was the tumor diameter (>4 cm) (Table 5). Finally, according to multivariate analysis, in the preoperative period, the presence of LVSI on the biopsy specimen and tumor diameter (>4 cm) were the independent risk factors for LNM, and in the postoperative period, the presence of LVSI on the biopsy specimen and parametrial involvement were the independent risk factors for LNM (Table 6).

**TABLE 5:** Univariate and multivariate analysis of the risk factors for parametrial involvement in patients with clinical stage IB–IIA cervical cancer.

	Univariate	*p*	Multivariate	*p*
	OR (95% CI)		OR (95% CI)	
Preoperative period				
Vaginal invasion	4.00 (1.10–14.80)	0.040	2.10 (1.10–4.00)	0.101
LVSI (+) (on the biopsy material)	2.40 (1.20–4.70)	0.080	2.20 (1.50–3.40)	0.141
Age (<40 years)	0.70 (0.40–1.30)	0.201	0.80 (0.40–1.60)	0.345
Squamous histology	0.60 (0.50–0.70)	0.070	0.70 (0.50–0.90)	0.110
Tumor diameter (>4 cm)	5.80 (2.20–14.20)	0.002	5.00 (2.40–10.60)	0.004
Postoperative period				
Lymph node metastasis	8.00 (2.90–22.50)	0.005	7.10 (2.40–20.50)	0.010
Depth stromal invasion	4.40 (1.90–11.10)	0.038	3.80 (2.10–7.00)	0.070
LVSI (+) (on the surgical specimen)	3.00 (1.50–5.90)	0.081	2.80 (2.00–3.70)	0.204

Abbreviations: CI, confidence interval; LVSI, lymphovascular space invasion; OR, odds ratio.

**TABLE 6:** Univariate and multivariate analysis of the risk factors for lymph node metastasis in patients with clinical stage IB–IIA cervical cancer.

	Univariate	*p*	Multivariate	*p*
	OR (95% CI)		OR (95% CI)	
Preoperative period				
Vaginal invasion	3.10 (1.10–10.10)	0.004	2.00 (1.20–3.70)	0.080
LVSI (+) (on the biopsy material)	6.20 (3.00–14.40)	0.023	5.40 (2.10–14.30)	0.040
Age (<40 years)	0.70 (0.60–0.80)	0.201	0.80 (0.50–1.30)	0.302
Squamous histology	0.40 (0.10–1.70)	0.060	0.50 (0.30–0.90)	0.108
Tumor diameter (>4 cm)	6.00 (3.20–11.60)	0.005	4.60 (2.30–9.10)	0.020
Postoperative period				
Parametrial involvement	7.10 (1.80–27.50)	0.001	6.70 (3.20–14.10)	0.034
Depth stromal invasion	5.10 (2.50–10.80)	0.003	4.30 (2.20–8.50)	0.071
LVSI (+) (on the surgical specimen)	7.80 (2.30–25.10)	0.004	6.40 (3.00–14.40)	0.0206

Abbreviations: CI, confidence interval; LVSI, lymphovascular space invasion; OR, odds ratio.

## 4 DISCUSSION

With this research, we implemented a retrospective analysis of 158 patients with ESCC who were treated with traditional (non‐nerve sparing) radical surgery at our reference center in Turkey. We observed that LNM was the only independent risk factor for regional and distant recurrence. However, there was no independent risk factor for local recurrence in patients with clinical stage IB–IIA cervical cancer. Furthermore, we have found that nearly perfect local (%99) control was achieved with traditional (non‐nerve sparing) radical hysterectomy in women with ESCC.

### 4.1 Is less radical surgery safe for patients with FIGO stage IB–IIA cervical cancer?

This issue remains a matter of debate. Many authors argue that nerve‐sparing radical hysterectomy or even simple hysterectomy is sufficient for the management of women with early clinical FIGO stage cervical cancer. The biggest concern is whether adequate local control can be achieved. Plante et al.^8^ found that in patients with stage IA2–IIB1 and low‐risk (limited stromal invasion and tumor diameter <2 cm) cervical cancer, simple hysterectomy was similar to radical hysterectomy in terms of pelvic recurrence at 3 years. However, that study included only certain low‐risk patients and most of the patients were diagnosed with loop electrosurgical excision procedure or conization, meaning that there was no tumor in these patients at the time of definitive surgical treatment; in other words, there was no risk of tumor cell spread during surgery. Bizzari et al.^13^ stated that non‐nerve‐sparing radical hysterectomy was related to a higher 5‐year DFS than less radical and nerve‐sparing radical hysterectomy for those with a tumor diameter of 20–40 mm. In line with Bizzari et al., our study showed a better local control rate and satisfactory 5‐year DFS and OS rates. Finally, in our opinion, although less radical surgery seems feasible for low‐risk patients, it does not seem safe for high‐risk patients. We attribute this better local control result to the removal of more parametrial or para‐uterine tissues in the surgery that we performed, thus removing possible microscopically involved areas with safe negative margins and reducing the risk of possible perineural invasion by removing the nerve tissues.

### 4.2 Does definitive surgical technique affect the pattern of recurrence?

This point is also controversial in the management of ESCC patients because in these women, OS is affected by all types of recurrence. Our study showed that all kinds of recurrence patterns affect OS, a similar finding to two previous studies.^17^, ^18^ Kato et al.^17^ argued that PI was an independent risk factor for local recurrence, vaginal invasion for regional recurrence, and LNM for distant metastasis. A more recent study stated that all types of recurrence were associated with LNM, large tumor diameter (>4 cm), and PI.^18^ However, in these two studies, which type of surgical technique was applied to which patient was unclear. Contrary to Kato's study, according to the multivariate analysis of our research, we have observed that vaginal invasion and PI were not independent risk factors for local, regional, or distant recurrence. During our study period, we detected only two local recurrences. Our study revealed that LNM was the only independent risk factor for regional and distant recurrences. We attribute these differences to the fact that we performed more radical surgery on all the patients; therefore, we believe that a radical approach (traditional non‐nerve‐sparing radical hysterectomy) is necessary, especially in patients with a high risk of PI or vaginal invasion. Furthermore, we found that OS was affected by recurrence and LNM, but the presence of vaginal invasion, PI, DSI, and LVSI did not affect the OS in these patients.

### 4.3 What are the independent risk factors for parametrial involvement and lymph node metastasis?

As a result of our study, the only independent risk factor for PI in the preoperative period in ESCC patients was the tumor diameter (>4 cm). Liang et al.^19^ reported that LNM, DSI, and LVSI (+) were independent risk factors for PI, and they argued that Stage IB cervical cancer patients with negative LNM or LVSI (−) and DSI negativity might be considered for less radical (Q‐M type B radical) hysterectomy. In another study, Kılıc et al.^12^ observed that LVSI (+), surgical margin positivity and LNM were independent risk factors for PI. This difference regarding independent risk factors of PI between our study and the aforenamed studies can be attributed to the fact that there was only one patient with a positive surgical margin in our search, and we evaluated a relatively limited number of patients compared to the other two studies.^12^, ^19^ Further, as a result of our research, the independent risk factors for LNM were described as LVSI (+), parametrial involvement, and large tumor diameter (>4 cm). These findings were similar to those of many previous studies. Sakuragi et al.^20^ stated that LNM risk increases with advanced FIGO stage and tumor size. Kato et al. reported that the risk of LNM was 7.4% for tumor diameters less than 2 cm and 22.2% for those with tumor diameters larger than 2 cm.^21^ Another study from Turkey observed that the tumor diameter and LVSI (+) (on the diagnostic specimen) were independent risk factors for LNM in the preoperative period.^22^ Thus, we also evaluated preoperative and postoperative parameters separately to identify preoperative high‐risk patients. In light of these findings, we believe that patients with ESCC should be observed carefully in the preoperative period. More radical surgery should be performed, especially if a high‐risk parameter (e.g., a tumor diameter >4 cm) is present.

The limitations of this research include its retrospective design, the inclusion of only ESCC (only clinical stage IB and IIA patients) patients, and the lack of evaluation of the intraoperative and postoperative complications. Another potential limitation is the lack of a comparative group of patients who underwent nerve‐sparing or minimally invasive surgery because only open the traditional radical type III hysterectomy technique was performed in our center during the study period. Despite these limitations, our study presents one of the largest and long‐term series of cases with ESCC. Further, our study includes patients who were operated on by the same experienced surgeons in a single referral center and who had a follow‐up period of 20 years. Therefore, the availability of good follow‐up data increased the precision of the results and offset the weaknesses of our study.

## 5 CONCLUSION

Traditional (non‐nerve sparing) radical surgery is safe and necessary to improve local control and decrease the local recurrence rate in women with ESCC. We observed that LNM was the only independent risk factor for regional and distant recurrences. However, there was no independent risk factor for local recurrence in patients with clinical stage IB–IIA cervical cancer.

## AUTHOR CONTRIBUTIONS

OA: operator, writing, design, statistical analysis, SA: operator, design, collection of data, FV: operator, TDY: pathologic evaluation, design CS: operator, writing, design, critical comments.

## FUNDING INFORMATION

The authors declare that no funds, grants, or other support were received during the preparation of this manuscript.

## CONFLICT OF INTEREST STATEMENT

The authors have no conflicts of interest to declare.

## DISCLOSURE

The authors declare that no funds, grants, or other support were received during the preparation of this manuscript.

## Data Availability

Research data are not shared.
